# Joint Effects of Socioeconomic Position, Race/Ethnicity, and Gender on COVID-19 Mortality among Working-Age Adults in the United States

**DOI:** 10.3390/ijerph19095479

**Published:** 2022-04-30

**Authors:** Elizabeth B. Pathak, Janelle M. Menard, Rebecca B. Garcia, Jason L. Salemi

**Affiliations:** 1 Women’s Institute for Independent Social Enquiry (WiiSE), Olney, MD 20832, USA; janelle.menard@wiise-usa.org (J.M.M.); becky.garcia@premisehealth.com (R.B.G.); 2 Premise Health, Brentwood, TN 37027, USA; 3 College of Public Health, University of South Florida, Tampa, FL 33620, USA; jsalemi@usf.edu

**Keywords:** COVID-19, mortality, social class, race/ethnicity, gender

## Abstract

Substantial racial/ethnic and gender disparities in COVID-19 mortality have been previously documented. However, few studies have investigated the impact of individual socioeconomic position (SEP) on these disparities. **Objectives:** To determine the joint effects of SEP, race/ethnicity, and gender on the burden of COVID-19 mortality. A secondary objective was to determine whether differences in opportunities for remote work were correlated with COVID-19 death rates for sociodemographic groups. **Design:** Annual mortality study which used a special government tabulation of 2020 COVID-19-related deaths stratified by decedents’ SEP (measured by educational attainment), gender, and race/ethnicity. **Setting:** United States in 2020. **Participants:** COVID-19 decedents aged 25 to 64 years old (*n* = 69,001). **Exposures:** Socioeconomic position (low, intermediate, and high), race/ethnicity (Hispanic, Black, Asian, Indigenous, multiracial, and non-Hispanic white), and gender (women and men). Detailed census data on occupations held by adults in 2020 in each of the 36 sociodemographic groups studied were used to quantify the possibility of remote work for each group. **Main Outcomes and Measures:** Age-adjusted COVID-19 death rates for 36 sociodemographic groups. Disparities were quantified by relative risks and 95% confidence intervals. High-SEP adults were the (low-risk) referent group for all relative risk calculations. **Results:** A higher proportion of Hispanics, Blacks, and Indigenous people were in a low SEP in 2020, compared with whites. COVID-19 mortality was five times higher for low vs. high-SEP adults (72.2 vs. 14.6 deaths per 100,000, RR = 4.94, 95% CI 4.82–5.05). The joint detriments of low SEP, Hispanic ethnicity, and male gender resulted in a COVID-19 death rate which was over 27 times higher (178.0 vs. 6.5 deaths/100,000, RR = 27.4, 95% CI 25.9–28.9) for low-SEP Hispanic men vs. high-SEP white women. In regression modeling, percent of the labor force in never remote jobs explained 72% of the variance in COVID-19 death rates. **Conclusions and Relevance:** SARS-CoV-2 infection control efforts should prioritize low-SEP adults (i.e., the working class), particularly the majority with “never remote” jobs characterized by inflexible and unsafe working conditions (i.e., blue collar, service, and retail sales workers).

## 1. Background

COVID-19 is a viral infectious disease with a simple etiology (infection with the novel coronavirus SARS-CoV-2) and a complex clinical course which encompasses pathological derangement of multiple organ systems (e.g., respiratory [1], vascular [2], neurological [3], endocrine [4], and reproductive [5]), both an acute (days to weeks) and chronic (months to >1 year) clinical phase [6,7], and as yet unknown long-term clinical sequelae. Human-to-human transmission of SARS-CoV-2 occurs via exhalation of viral-laden aerosols by an infected person, suspension of these viral-laden aerosols in ambient air for extended periods of time, travel on expiratory plumes, and inhalation by susceptible persons at both near-field and far-field distances [8,9,10]. Put simply, the social environments which can lead to SARS-CoV-2 infection are those in which people are breathing other people’s breath [11,12].

For individuals, the accumulation of economic and social capital gives rise to a privileged socioeconomic position, which grants power relative to others in society [13]. This power manifests itself through control over economic resources (including labor and the means of production) and in a high social status that opens access to less tangible privileges via well-resourced social networks [14]. Numerous studies have verified the better health and lower mortality experienced by those with socioeconomic power and privilege [15,16,17,18,19,20]. Conversely, populations with low socioeconomic resources (i.e., the poor and working class) have historically experienced disproportionate exposure risks and burden of disease [21,22,23].

In the case of COVID-19, socioeconomic resources and privileges create the flexibility and space for the deployment of multiple strategies to reduce and prevent exposure to the highly infectious airborne novel coronavirus SARS-CoV-2. People in privileged socioeconomic positions live in larger homes with fewer people and in less densely populated neighborhoods (whether horizontally spacious in the suburbs or vertically spacious in metropolitan downtown areas), and rarely use public transportation. Additionally, the upper and professional classes have ready access to both high-quality outpatient health care and the best tertiary care hospital centers [24]. College education and related forms of social capital facilitate navigation of a complex health care system [25].

The vast majority of low-SEP adults are employed in blue collar, service, or retail sales jobs (i.e., working-class jobs) which require onsite attendance and prolonged close contact with others. In addition, working conditions vary by gender [26] and race/ethnicity as well as SEP [19,27]. The most physically hazardous occupations are highly segregated by gender and performed largely by men (e.g., meatpacking). At the same time, under racialized capitalism, whites enjoy advantages of occupational status even within narrowly defined job categories, compared with Hispanic, Black, and Indigenous workers with comparable educational credentials [27,28,29]. Moreover, elevated infection risks are amplified across multiple social environmental scales for working-class adults [30], who may reside in poorly ventilated housing [31], commute in a crowded carpool, and labor in a crowded, poorly ventilated worksite.

Our study is a national investigation of the joint effects of socioeconomic position (SEP), gender, and race/ethnicity on COVID-19 mortality in working-age adults, and it takes advantage of an ad hoc death certificate tabulation released by the U.S. National Center for Health Statistics (NCHS) in February 2021 [32,33]. These data permitted the calculation of age-adjusted COVID-19 mortality rates stratified simultaneously by socioeconomic position, race/ethnicity, and gender.

We hypothesized that there were (1) significant SEP disparities in COVID-19 mortality in working-age adults; (2) significant SEP disparities in every racial/ethnic group; and (3) within-SEP strata gender and racial/ethnic differences in opportunities for remote work that would be correlated with within- and between-SEP strata gender and racial/ethnic disparities in COVID-19 mortality.

## 2. Data and Methods

### 2.1. Population at Risk

Our target population included adults aged 25 to 64 years who were U.S. residents during 2020. We included six racial/ethnic groups: whites, Hispanics, Blacks, Asians, Indigenous, and multirace. The Indigenous group included American Indians, Alaska Natives, Native Hawaiians, and other Pacific Islanders, who were grouped together because of small numbers of deaths in some age-social class strata.

Reductionist narratives of race and cultural, moral, and biological inferiority [34,35] persist in public health and medicine when race is cited as an explanatory variable for negative health outcomes in the absence of social and historical contexts. In opposition to these biased approaches, we take an explicitly anti-essentialist stance [36] on the meaning of race and ethnicity [37] in the epidemiology of COVID-19. We recognize race not as a genetic or physiological risk factor, but rather as a social construct [38] that is embedded within a nexus of social oppression, exploitation, and conflict. This nexus amplifies exposure risks that result in higher burdens of morbidity and mortality among racial and ethnic minority populations.

### 2.2. Measurement of Socioeconomic Position

As observed nearly 40 years ago, the association between low socioeconomic resources and adverse health outcomes is robust regardless of the specific measures employed in individual studies [22]. Educational attainment is frequently used in the United States as a measure of socioeconomic position because it is more widely collected and recorded than occupation or annual income [39]. Furthermore, educational attainment is a more stable indicator of SEP over time, and is far less likely to be missing or unknown for women than occupation. The National Center for Health Statistics (NCHS) added educational attainment to the 1989 revision of the model death certificate specifically as “as a more reliable measure of socioeconomic status than occupation” [40]. Furthermore, Krieger et al. (1997) prefer the term “socioeconomic position” (as opposed to “socioeconomic status”) for measures which capture actual resources (e.g., a college degree) vs. relative prestige [39]. We used educational attainment data to define three ordinal strata of SEP, with consideration of credentialism as an important mechanism by which education conveys health benefits in society [39]. **Low-SEP** adults had no education beyond high school. The vast majority of adults aged 25–64 years in this group had graduated from high school. **Intermediate-SEP** adults had at least one year of college attendance, but did not have a 4-year college degree (bachelor’s degree). This stratum included those with associate’s degrees, and other technical/vocational certifications (e.g., licensed practical nurses). **High-SEP** **adults** had at minimum a bachelor’s degree. This stratum included everyone with advanced degrees. We chose not to further stratify “very low SEP” (i.e., those without a high school diploma) or “very high SEP” (i.e., those with Master’s and Doctoral degrees) because of the need to maintain sufficient cell size counts to support our plan to further divide each SEP stratum 36 times for the purpose of age adjustment.

### 2.3. COVID-19 Deaths

**COVID-19-involved deaths** included all deaths for which COVID-19 (ICD-10 code U07.1) was listed as the underlying or a contributing cause of death on the death certificate. We analyzed provisional death counts for 2020 stratified by four sociodemographic variables: (1) educational attainment (no college, some college, and college graduate); (2) race and ethnicity (white non-Hispanic, Hispanic, Black non-Hispanic, Asian non-Hispanic, American Indian/Alaska Native non-Hispanic, Native Hawaiian and other Pacific Islander non-Hispanic, more than one race non-Hispanic, and unknown); (3) gender (male, female, and unknown); and (4) age group (25–39 years, 40–54 years, and 55–64 years) [32,33].

### 2.4. Population Denominators

We used the 2020 Annual Social and Economic Supplement (ASEC) to the Current Population Survey (CPS) to calculate national population estimates stratified by educational attainment, race/ethnicity, gender, and age to exactly match the strata available in the COVID-19 death dataset [41]. Public-use CPS datasets include statistical weights to calculate national population estimates from the household-based sample [42]. We used special alternative weights that compensated for lower 2020 response rates in the CPS which were found to be differential by respondent income [42,43].

### 2.5. Death Rate Calculations

We first calculated age-specific death rates (deaths/population) for three age strata (25–39 years, 40–54 years, and 55–64 years) by SEP for the following groups: (a) all adults combined; (b) men and women; (c) six racial/ethnic groups, and (d) 12 groups defined by both gender and race/ethnicity. Next, we verified that SEP patterns of mortality were similar across age for all population groups. Then, we calculated age-adjusted mortality rates for ages 25–64 combined, using the direct method with the U.S. 2020 population as the standard.

### 2.6. Socioeconomic Position and Occupation Distributions

For the 36 sociodemographic groups aged 25 to 64 years (3 SEP strata × 2 gender strata × 6 race/ethnicity strata), we used the 2020 CPS ASEC [41] to identify the percent of adults with reported occupation in the following mutually exclusive categories: (1) blue collar, (2) service, (3) retail sales, (4) health professionals, and (5) white collar (excluding health professionals and retail sales). Further details and specific examples of common job titles in each of these categories can be found in Table S1.

We rated each job title in the CPS on its potential for remote work (i.e., work from home). All blue collar, service, and retail sales jobs were classified as “never remote” jobs. All other jobs were classified as “sometimes remote” (health professionals) or “feasibly remote” (all other white collar jobs).

### 2.7. Analytic Methods

We calculated SEP rate ratios (RRs) of the age-adjusted death rates for the entire study population, by gender, by race/ethnicity, and finally by gender and race/ethnicity simultaneously. High-SEP individuals (college graduates) were the referent group for all comparisons. Then, we calculated disparity RRs that compared COVID-19 mortality in 35 sociodemographic groups with a single low-risk referent group (high-SEP white women). Finally, we regressed the population-weighted log-transformed age-adjusted COVID-19 mortality rates against the percent of workers employed in never remote jobs for the 36 sociodemographic groups described above.

## 3. Results

**Figure 1:**
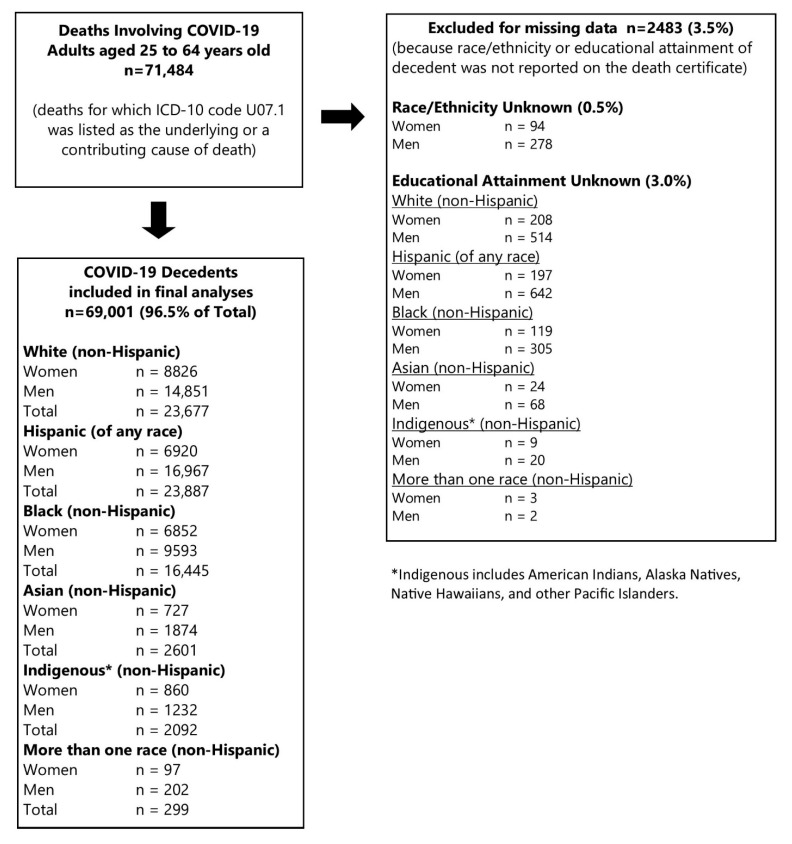
Study Inclusion of Deaths Involving COVID-19 in Adults Aged 25–64 Years Old, United States, 1 January 2020 to 31 December 2020.

There were 71,484 COVID-19-involved deaths among adults aged 25 to 64 years old during calendar year 2020 (Figure 1), as reported to NCHS by the end of February 2021. There were very few missing data; 2483 deaths (3.5%) were excluded for missing race/ethnicity (0.5%) or educational attainment (3.0%). The final analytic dataset included 96.5% of the total deaths (*n* = 69,001) (Figure 1).

### 3.1. Socioeconomic Position Distribution of the Population at Risk

**Figure 2:**
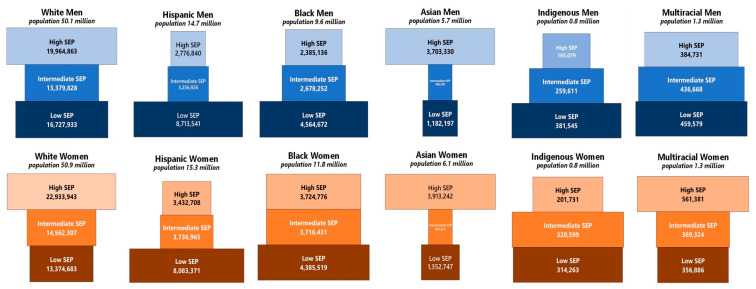
Socioeconomic Position (SEP) Population Pyramids * among Adults 25–64 Years Old (*n* = 168.4 million), United States 2020. **Note**: * The width of each bar is proportional to the size of the social class stratum within each race/ethnicity-gender group. Indigenous includes American Indians, Alaska Natives, Native Hawaiians, and other Pacific Islanders.

There were 168.4 million adults aged 25 to 64 years old in the U.S. in 2020. Figure 2 presents SEP population pyramids for each of the 12 gender-race/ethnicity groups. In each pyramid, high-SEP adults are represented in the top tier, intermediate-SEP adults in the middle tier, and low-SEP adults (i.e., the working class) in the bottom tier. White men and women comprised approximately 60.2% of the total population at risk for COVID-19 mortality in working-age adults, and high SEP comprised the largest class among whites. Hispanics were predominantly of low SEP. Low SEP also predominated among Black and Indigenous men.

### 3.2. Socioeconomic Position and COVID-19 Mortality: Total Population

The age-adjusted COVID-19 mortality rate among high-SEP adults aged 25–64 years was 14.6 deaths per 100,000 (Table 1). The death rate was twice as high among intermediate-SEP adults (30.4 deaths/100,000; RR = 2.08, 95% CI 2.02–2.14) and five times as high among low-SEP working-class adults (72.2 deaths/100,000; RR = 4.94, 95% CI 4.82–5.05). The majority (68%, *n* = 46,966) of COVID-19 decedents were in a low SEP, and only 12% (*n* = 8421) had a high SEP.

### 3.3. Socioeconomic Position and COVID-19 Mortality by Gender

Women experienced lower COVID-19 death rates than men (high-SEP women: 10.0 deaths/100,000 vs. 19.8 deaths/100,000 in high-SEP men), but a slightly higher disparity for low vs. high SEP (RR = 5.06, 95% CI 4.87–5.26 in women vs. RR = 4.65, 95% CI 4.52–4.79 in men). Numerically, both the age-adjusted death rate (92.1/100,000) and the number of deaths (*n* = 31,258) were highest for low-SEP men (Table 1).

### 3.4. Socioeconomic Position and COVID-19 Mortality by Race and Hispanic Ethnicity

In all six racial/ethnic groups, there was a monotonic association between SEP and COVID-19 mortality, with the lowest age-adjusted death rates in high-SEP adults, and the highest rates in low-SEP adults (Table 1). SEP disparity RRs ranged from 2.18 (95% CI 2.00–2.38) among Asians to RR = 4.90 (95% CI 4.11–5.84) among Indigenous adults. Within each stratum of SEP, death rates were highest for Indigenous, Hispanic, and Black adults, and lowest for multiracial, Asian, and white adults.

### 3.5. Disparities in COVID-19 Mortality: Independent and Joint Effects of Socioeconomic Position, Gender, and Race/Ethnicity

**Figure 3:**
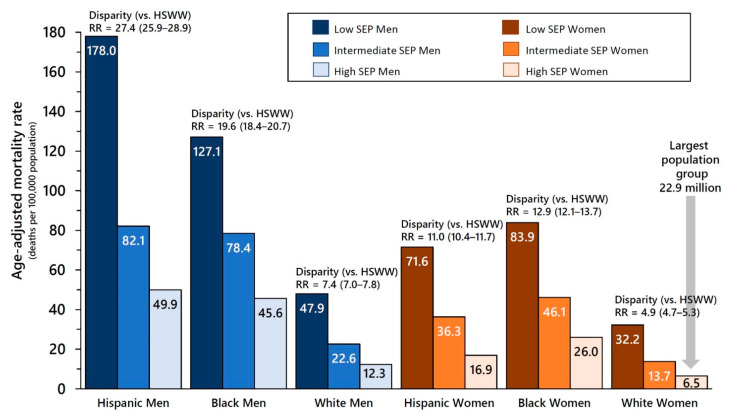
COVID-19 Death Rates by Socioeconomic Position (SEP), Gender, and Race/Ethnicity with High-SEP White Women (HSWW) as the Referent Group for Disparity Rate Ratios ** among Adults 25–64 Years Old, United States 1 January 2020 to 31 December 2020. **Note**: ** The disparity rate ratios (RR) are calculated separately for each sociodemographic group and compare age-adjusted COVID-19 death rates to the referent group (high-SEP white women (HSWW)). Results are presented for the three largest population groups (whites, Hispanics, and Blacks). All rate ratios and full results for Asians, Indigenous adults (American Indians, Alaska Natives, Native Hawaiians, and other Pacific Islanders) and multiracial adults are shown in Table S2.

The independent effects of SEP, gender, and race/ethnicity on COVID-19 mortality are evident in Figure 3 for Hispanics, Blacks, and whites, who together comprised 90.5% of the total population at risk. Across all six groups defined by gender and race/ethnicity, there was a strong and statistically significant association of SEP with age-adjusted COVID-19 mortality (see Table S2 for all RRs and 95% CI). Similarly, across all nine groups defined by SEP and race/ethnicity, age-adjusted death rates were always higher for men than for women. However, there was effect modification by gender when stratifying by SEP. Within each of the three SEP strata, the highest death rates were suffered by Hispanics among men, and by Blacks among women.

Finally, disparity RRs which capture the joint effects of SEP, gender, and race/ethnicity on COVID-19 mortality in working-age adults confirm that high-SEP white women were at lowest risk for COVID-19 mortality (6.5 deaths/100,000). The joint detriments of low (i.e., working class) SEP, Hispanic ethnicity, and male gender resulted in a COVID-19 age-adjusted death rate which was over 27 times higher (178.0 deaths/100,000, RR = 27.4, 95% CI 25.9–28.9) compared with high-SEP white women (Figure 3). While in all SEP strata Hispanic and Black women experienced lower death rates than Hispanic and Black men, respectively, they still suffered higher death rates than white men. Full results for all 36 sociodemographic groups are available in Table S2.

### 3.6. Working-Class Jobs and Never Remote Work by Socioeconomic Position, Gender, and Race/Ethnicity

**Figure 4:**
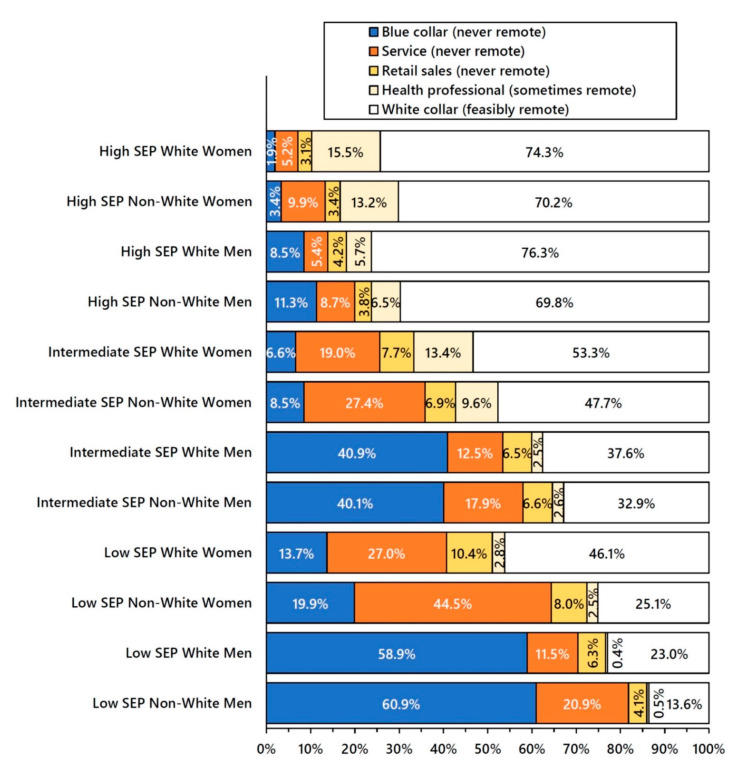
Remote Work Occupations * by Socioeconomic Position (SEP), Gender, and Race/Ethnicity # among Adults 25–64 Years Old, United States 2020. **Note**: * The denominators for occupation percentages include only persons who were in the labor force with a reported occupation in the Current Population Survey (CPS), the representative sample from which national population estimates were derived. Service occupations include health care support, protective service, food service, housekeeping, building and grounds, and personal care service workers. Registered nurses and licensed practical nurses are classified as health professionals. Transportation workers, including airline pilots and flight attendants, are classified as blue collar. White collar feasibly remote is comprised of managers, professionals, technical workers, non-retail sales workers, and office support and administrative workers. # Non-white includes Hispanic, Black, Asian, Indigenous, and multiracial adults.

As predicted, the majority of high-SEP adults had white collar jobs, and those of intermediate SEP were employed in a mixture of blue collar, service, retail sales, and white collar jobs, with no category in the majority (Figure 4). Conversely, the majority of low-SEP adults were employed in working-class jobs (blue collar, service, and retail sales) with no potential for remote work. However, majority employment in working-class jobs varied from 51.1% of low-SEP white women to 85.9% of low-SEP non-white men. In all SEP strata, non-whites were more likely to be employed in service jobs than whites, and men were much more likely to be employed in blue collar jobs than women.

**Figure 5:**
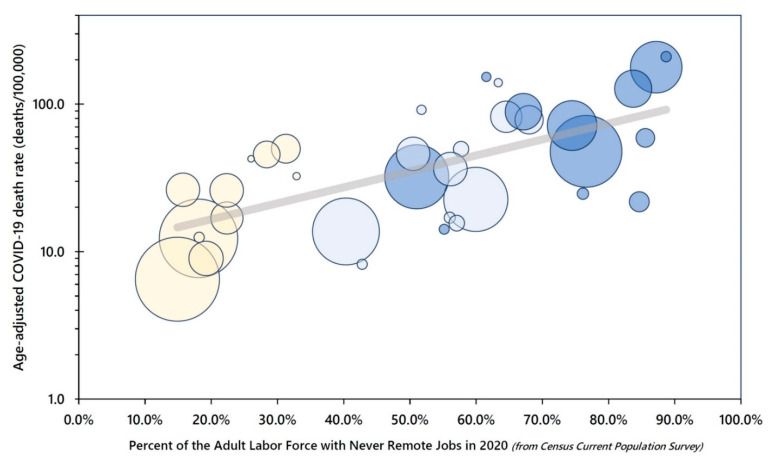
COVID-19 Mortality by Never Remote (Blue Collar/Service/Retail) Jobs among Adults 25 to 64 Years Old, United States 2020. **Note**: Each bubble represents a socioeconomic position (SEP)-gender-racial/ethnic group, with bubble size proportional to population size. Dark blue = low SEP, light blue = intermediate SEP, and yellow = high SEP.

A population-weighted regression of the natural log-transformed age-adjusted 2020 COVID-19 death rates revealed a good fit of an exponential model in which the percent of adults employed in never remote jobs during 2020 explained 72% of the variance in the age-adjusted death rates across the 36 population groups defined by socioeconomic position, gender, and race/ethnicity (Figure 5).

## 4. Discussion

People with high socioeconomic positions retain a far greater degree of discretionary control over their professions, work lives, and daily schedules than workers of low SEP. For many, a college degree and professional status permits a measure of autonomy and flexibility in meeting job requirements [44]. In contrast, the working class (in blue collar, service, and retail sales occupations) are subjected to authoritarian control [45] and inflexible requirements of work [17,27,44,46]. Moreover, the worksites in which the working class perform their wage labor are often replete with physical, chemical, and biological hazards which directly and negatively impact workers’ health and well-being [44,47,48,49]. The results of our census data analyses confirm that educational attainment is highly correlated with occupational segregation, with the majority of low-SEP adults in working-class jobs (i.e., blue collar, service, and retail sales) across all gender-race/ethnicity groups.

In the United States, individual socioeconomic position results from an intrinsically racialized set of economic and social status relationships [50,51,52]. The legacies of colonialism, slavery, and other forms of structural racism shape local labor markets, housing opportunities, and other material aspects of workers’ lives [19,53]. Consequently, compared with whites, a given level of educational attainment usually provides fewer economic benefits to Blacks and other minorities [19,53].

Our results support the hypothesis that hazardous conditions of work were a primary driver of joint socioeconomic, gender, and racial/ethnic disparities in COVID-19 mortality. During the first year of the COVID-19 pandemic in the United States, low-SEP adults aged 25–64 years old were five times as likely as high-SEP adults to die from COVID-19, and intermediate-SEP adults were twice as likely as high-SEP adults to die. High-SEP whites aged 25 to 64 years were largely shielded from COVID-19 mortality during the first year of the pandemic. They comprised more than one-quarter of the study population, but accounted for only 5% of the COVID-19 deaths. High-SEP white women, the numerically largest population group (*n* = 22.9 million), accounted for only 2% of COVID-19 decedents in working-age adults. In contrast, Hispanic and Black low-SEP (i.e., working-class) men comprised only 8% of the 25–64 years old population, but they were 29% of the premature COVID-19 decedents. Non-white low-SEP men were most likely to be employed in never remote occupations (i.e., blue collar, service, and retail sales) compared with every other sociodemographic group.

Our results are consistent with those of a smaller study of excess mortality by occupation in California during the period March–October 2020 [54], and with a small study of worksite COVID-19 transmission in Asian countries which found the most commonly affected occupations were health care, drivers, sales, cleaners, and public safety [55]. A major report on social inequalities in COVID-19 in the United Kingdom found social class patterns of COVID-19 mortality that were very similar to what we observed for the U.S. [31]. However, the magnitude of the socioeconomic mortality disparities was much lower in the U.K.

### 4.1. COVID-19 Case Fatality

Axiomatically, mortality rates (deaths/population) are a function of two underlying phenomena: the incidence of disease in a specified population (cases/population) and the case fatality rate (deaths/cases) of the disease. We hypothesize that disparities in both case fatality and incidence have contributed to the strong and highly significant mortality disparities observed in our study. Access to high-quality evidence-based medical care is not universal in the U.S. [56]. Barriers to accessing timely and appropriate COVID-19 medical care include lack of health insurance, inadequate health insurance (e.g., high deductible/co-pay plans), lack of or inadequate paid sick leave [57], geographic location, transportation access/costs/timeliness, lack of respite dependent care, threat of job loss, immigration status, racism and discrimination, and distrust of health care and government institutions [58]. An analysis of place of death of U.S. COVID-19 decedents found that 22% of 30–49 year olds and 14% of 50–64 year olds died either outside a hospital or in the emergency department (OH/ED) [59]. Minimizing COVID-19 case fatality requires that individuals have access to timely diagnosis and high-quality hospital medical care before they become critically ill.

### 4.2. Study Limitations and Public Health Data Gaps

It is likely that COVID-19 deaths in the U.S. have been undercounted (i.e., cause of death has been misclassified), and this misclassification is likely to be differential by socioeconomic position, resulting in a bias toward the null in our estimates of socioeconomic disparities. Misclassification occurs when there is insufficient medical information available at the time of death. Lack of access to medical care and out-of-hospital mortality can result in the use of non-specific cause of death coding on death certificates. We have previously shown that the percent of all non-injury deaths coded to “symptoms, signs, and ill-defined conditions” increased from 2019 to 2020 among working-age adults [59].

A simple step toward improving COVID-19 surveillance data, which could be implemented immediately across a wide range of data systems, is to add one yes/no question to all individual adult patient encounter medical records: “Has this person completed one or more years of college?” A “no” response on this single data item would identify low-SEP adults (i.e., the working class). A follow-up question for those who replied “yes” (“Does this person have a 4-year college degree?”) would easily differentiate intermediate- vs. high-SEP adults.

## 5. Conclusions

The most urgent implication of our study points to immediate actions needed to protect low-SEP adults, particularly blue collar, service, and retail sales workers, from infection with the SARS-CoV-2 virus. Expert recommendations include strengthening federal and state labor laws [60], empowering OSHA [49], adopting the Total Worker Health Framework [61], and direct actions for unions to organize for greater protections for worker safety [48].

## Figures and Tables

**Table 1:** Socioeconomic Position (SEP) Disparities in Reported COVID-19 Mortality Overall and by Gender and Race/Ethnicity among Adults 25–64 Years Old in the United States, 2020.

Demographic Groups	Low SEP	Intermediate SEP	High SEP
Population at risk	59.9 million	44.4 million	64.1 million
**Total Study Population**			
COVID-19 deaths	46,966	13,614	8421
Age-adjusted mortality rate	72.2/100,000	30.4/100,000	14.6/100,000
SEP rate ratio (95% CI)	4.94 (4.82–5.05)	2.08 (2.02–2.14)	1.0 (referent)
**By Reported Gender**			
**Women**			
COVID-19 deaths	15,708	5535	3039
Age-adjusted mortality rate	50.4/100,000	22.8/100,000	10.0/100,000
SEP rate ratio (95% CI)	5.06 (4.87–5.26)	2.29 (2.19–2.39)	1.0 (referent)
**Men**			
COVID-19 deaths	31,258	8079	5382
Age-adjusted mortality rate	92.1/100,000	39.5/100,000	19.8/100,000
SEP rate ratio (95% CI)	4.65 (4.52–4.79)	1.99 (1.93–2.06)	1.0 (referent)
**By Reported Race/Ethnicity**			
**White, non-Hispanic**			
COVID-19 deaths	14,587	5344	3746
Age-adjusted mortality rate	40.6/100,000	17.8/100,000	9.3/100,000
SEP rate ratio (95% CI)	4.37 (4.21–4.53)	1.92 (1.84–2.00)	1.0 (referent)
**Hispanic**			
COVID-19 deaths	19,174	3173	1540
Age-adjusted mortality rate	125.0/100,000	57.0/100,000	32.9/100,000
SEP rate ratio (95% CI)	3.80 (3.61–4.00)	1.73 (1.63–1.84)	1.0 (referent)
**Black, non-Hispanic**			
COVID-19 deaths	10,544	3912	1989
Age-adjusted mortality rate	105.9/100,000	59.0/100,000	33.8/100,000
SEP rate ratio (95% CI)	3.14 (2.99–3.29)	1.75 (1.66–1.84)	1.0 (referent)
**Asian, non-Hispanic**			
COVID-19 deaths	1149	497	955
Age-adjusted mortality rate	38.5/100,000	32.1/100,000	17.7/100,000
SEP rate ratio (95% CI)	2.18 (2.00–2.38)	1.82 (1.63–2.03)	1.0 (referent)
**Indigenous, non-Hispanic**			
COVID-19 deaths	1353	602	137
Age-adjusted mortality rate	182.1/100,000	113.4/100,000	37.2/100,000
SEP rate ratio (95% CI)	4.90 (4.11–5.84)	3.05 (2.53–3.67)	1.0 (referent)
**Multirace/Other, non-Hispanic**			
COVID-19 deaths	159	86	54
Age-adjusted mortality rate	20.0/100,000	12.9/100,000	8.7/100,000
SEP rate ratio (95% CI)	2.32 (1.70–3.15)	1.49 (1.06–2.10)	1.0 (referent)

## Data Availability

The publicly available datasets analyzed in this study are referenced in the Methods section of this paper.

## Supplementary Materials

The following supporting information can be downloaded at: https://www.mdpi.com/article/10.3390/ijerph19095479/s1, Table S1: Top Five Occupations of Adults 25–64 years old in 2020, by Socioeconomic Position, Gender, and Race/Ethnicity. Table S2: Disparities in COVID-19-Related Mortality by Socioeconomic Position, Race/Ethnicity, and Gender Among Adults 25–64 Year Old in the United States, 1 January 2020 to 31 December 2020.
