# Supplementary material for: Joint Effects of Socioeconomic Position, Race/Ethnicity, and Gender on COVID-19 Mortality among Working-Age Adults in the United States
Source: Int J Environ Res Public Health. 2022 Apr 30;19(9):5479. doi: 10.3390/ijerph19095479 (PMC9102098; doi:10.3390/ijerph19095479)
Supplement: Supplementary file 1 [file ijerph-19-05479-s001.zip › Pathak Table S1 FINAL REVISED.pdf]

Table S1. Top Five Occupations of Adults 25–64 years old in 2020, by Socioeconomic Position, Gender, and Race/Ethnicity. **Blue** = Blue collar, never remote **Orange** = Service, never remote; **Gold** = Retail sales, never remote; **Yellow** = Health professionals, rarely remote; **White** = White collar, feasibly remote.

Demographic Group	Low Socioeconomic Position	Intermediate Socioeconomic Position	High Socioeconomic Position
**White men**	1. Driver/sales workers, and truck drivers<br>2. Construction laborers<br>3. Managers, other<br>4. Carpenters<br>5. Laborers, hand material movers	1. Managers, other<br>2. Driver/sales workers, and truck drivers<br>3. Retail supervisors<br>4. Electricians<br>5. Retail salespersons	1. Managers, other<br>2. Software developers<br>3. Chief executives, legislators<br>4. Lawyers, judges, magistrates<br>5. Elementary, middle school teachers
**Hispanic men**	1. Construction laborers<br>2. Driver/sales workers, and truck drivers<br>3. Landscaping, grounds maintenance workers<br>4. Carpenters<br>5. Laborers, hand material movers	1. Driver/sales workers, and truck drivers<br>2. Construction laborers<br>3. Retail salespersons<br>4. Managers, other<br>5. Retail supervisors	1. Managers, other<br>2. Elementary, middle school teachers<br>3. Software developers<br>4. Accountants and auditors<br>5. Computer occupations, all other
**Black men**	1. Driver/sales workers, and truck drivers<br>2. Laborers, hand material movers<br>3. Janitors and building cleaners<br>4. Cooks<br>5. Construction laborers	1. Driver/sales workers, and truck drivers<br>2. Laborers, hand material movers<br>3. Retail salespersons<br>4. Security guards, gaming surveillance officers<br>5. Stockers and order fillers	1. Managers, other<br>2. Elementary, middle school teachers<br>3. Software developers<br>4. Driver/sales workers, truck drivers<br>5. Accountants and auditors
**White women**	1. Secretaries and administrative assistants<br>2. Retail supervisors<br>3. Cashiers<br>4. Bookkeeping and accounting clerks<br>5. Customer service representatives	1. Registered nurses<br>2. Secretaries and administrative assistants<br>3. Retail supervisors<br>4. Bookkeeping and accounting clerks<br>5. Managers, other	1. Elementary/middle school teachers<br>2. Registered nurses<br>3. Managers, other<br>4. Accountants and auditors<br>5. Secondary school teachers
**Hispanic women**	1. Maids and housekeepers<br>2. Janitors and building cleaners<br>3. Cooks<br>4. Personal care aides<br>5. Cashiers	1. Secretaries, administrative assistants<br>2. Cashiers<br>3. Office clerks, general<br>4. Customer service representatives<br>5. Bookkeeping and accounting clerks	1. Elementary/middle school teachers<br>2. Registered nurses<br>3. Managers, other<br>4. Education administrators<br>5. Secretaries, administrative assistants
**Black women**	1. Nursing assistants<br>2. Maids and housekeepers<br>3. Personal care aides<br>4. Cashiers<br>5. Home health aides	1. Nursing assistants<br>2. Customer service representatives<br>3. Registered nurses<br>4. Licensed practical nurses<br>5. Secretaries, administrative assistants	1. Registered nurses<br>2. Elementary/middle school teachers<br>3. Managers, other<br>4. Social workers<br>5. Accountants and auditors

NOTES: Current or most recent occupation was recorded in the census data only for adults who were in the labor force. Blue collar included construction, mining, farming, forestry, installation, maintenance, and repair jobs, fabrication and production (i.e., manufacturing) jobs, transportation, warehousing, material moving, and general manual labor jobs. Service jobs included housekeeping, janitorial, groundskeeping, food service, protective services, personal care, childcare, and healthcare service. White collar jobs included all managerial, professional (except health professionals), technical, sales (except retail sales), and administrative and office support occupations.
