# Supplementary material for: Joint Effects of Socioeconomic Position, Race/Ethnicity, and Gender on COVID-19 Mortality among Working-Age Adults in the United States
Source: Int J Environ Res Public Health. 2022 Apr 30;19(9):5479. doi: 10.3390/ijerph19095479 (PMC9102098; doi:10.3390/ijerph19095479)
Supplement: Supplementary file 1 [file ijerph-19-05479-s001.zip › Pathak Table S2 FINAL REVISED.pdf]

Table S2. Disparities in COVID-19-Related Mortality by Socioeconomic Position, [1] Race/Ethnicity, and Gender Among Adults 25–64 Year Old in the United States, 1 January 2020 to 31 December 2020.

Demographic Groups	Low SEP	Intermediate SEP	High SEP
**White Women**			
Population in 2020	13,374,683	14,562,307	22,933,943
COVID-19 deaths	5,291	2,177	1,358
Age-adjusted^ mortality rate	32.2/100,000	13.7/100,000	6.5/100,000
Disparity Rate Ratio (95% CI)#	4.9 (4.7-5.3)	2.1 (2.0-2.3)	1.0 (referent)
**White Men**			
Population in 2020	16,727,933	13,379,828	19,964,863
COVID-19 deaths	9,296	3,167	2,388
Age-adjusted^ mortality rate	47.9/100,000	22.6/100,000	12.3/100,000
Disparity Rate Ratio (95% CI) #	7.4 (7.0-7.8)	3.5 (3.3-3.7)	1.9 (1.8-2.0)
**Hispanic Women**			
Population in 2020	8,083,371	3,736,965	3,432,708
COVID-19 deaths	5,395	1,106	419
Age-adjusted^ mortality rate	71.6/100,000	36.3/100,000	16.9/100,000
Disparity Rate Ratio (95% CI) #	11.0 (10.4-11.7)	5.6 (5.2-6.1)	2.6 (2.3-2.9)
**Hispanic Men**			
Population in 2020	8,713,541	3,256,926	2,776,840
COVID-19 deaths	13,779	2,067	1,121
Age-adjusted^ mortality rate	178.0/100,000	82.1/100,000	49.9/100,000
Disparity Rate Ratio (95% CI) #	27.4 (25.9-28.9)	12.6 (11.8-13.5)	7.7 (7.1-8.3)
**Black Women**			
Population in 2020	4,385,519	3,716,431	3,724,776
COVID-19 deaths	4,091	1,833	928
Age-adjusted^ mortality rate	83.9/100,000	46.1/100,000	26.0/100,000
Disparity Rate Ratio (95% CI) #	12.9 (12.1-13.7)	7.1 (6.6-7.6)	4.0 (3.7-4.4)
**Black Men**			
Population in 2020	4,564,672	2,678,252	2,385,136
COVID-19 deaths	6,453	2,079	1,061
Age-adjusted^ mortality rate	127.1/100,000	78.4/100,000	45.6/100,000
Disparity Rate Ratio (95% CI) #	19.6 (18.4-20.7)	12.1 (11.3-12.9)	7.0 (6.5-7.6)
**Asian Women**			
Population in 2020	1,352,747	847,927	3,913,242
COVID-19 deaths	352	125	250
Age-adjusted^ mortality rate	21.8/100,000	15.6/100,000	9.0/100,000
Disparity Rate Ratio (95% CI) #	3.4 (3.0-3.8)	2.4 (2.0-2.9)	1.4 (1.2-1.6)
**Asian Men**			
Population in 2020	1,182,197	806,299	3,703,330
COVID-19 deaths	797	372	705
Age-adjusted^ mortality rate	59.1/100,000	49.6/100,000	26.3/100,000
Disparity Rate Ratio (95% CI) #	9.1 (8.3-9.9)	7.6 (6.8-8.6)	4.1 (3.7-4.4)
**Indigenous* Women**			
Population in 2020	314,263	320,599	201,731
COVID-19 deaths	526	269	65
Age-adjusted^ mortality rate	153.0/100,000	91.6/100,000	32.5/100,000

Demographic Groups	Low SEP	Intermediate SEP	High SEP
Disparity Rate Ratio (95% CI) #	23.5 (21.3-26.0)	14.1 (12.4-16.1)	5.0 (3.9-6.4)
**Indigenous* Men**			
Population in 2020	381,545	259,611	165,079
COVID-19 deaths	827	333	72
Age-adjusted^ mortality rate	210.0/100,000	139.9/100,000	42.6/100,000
Disparity Rate Ratio (95% CI) #	32.3 (29.6-35.2)	21.5 (19.1-24.3)	6.6 (5.2-8.3)
**Multiracial+ Women**			
Population in 2020	356,886	369,324	561,381
COVID-19 deaths	53	25	19
Age-adjusted^ mortality rate	14.2/100,000	8.2/100,000	insufficient data
Disparity Rate Ratio (95% CI) #	2.2 (1.7-2.9)	1.3 (0.8-1.9)	insufficient data
**Multiracial+ Men**			
Population in 2020	459,579	436,668	384,731
COVID-19 deaths	106	61	35
Age-adjusted^ mortality rate	24.7/100,000	17.0/100,000	12.5/100,000
Disparity Rate Ratio (95% CI) #	3.8 (3.1-4.6)	2.6 (2.0-3.4)	1.9 (1.4-2.7)

1 High SEP = college graduates; Intermediate SEP = some college; Low SEP = no college.

^ Age-adjusted using the direct method to the 2020 USA population. Age strata were 25–39 years, 40–54 years, and 55–64 years.

# The referent group for all disparity rate ratios were High SEP white women.

* Indigenous includes American Indians, Alaska Natives, Native Hawaiians, and other Pacific Islanders.

+ Non-Hispanic with more than one race reported on the death certificate. In the census (the source of population denominators) up to 5 races could be reported for an individual. The most numerous groups were White + Black and White + Asian.
